# Reduction of cardiovascular risk factors among young men with hypertension using an interactive decision aid: cluster-randomized control trial

**DOI:** 10.1186/s12872-021-02339-1

**Published:** 2021-11-16

**Authors:** Liina Kask-Flight, Koray Durak, Kadri Suija, Anneli Rätsep, Ruth Kalda

**Affiliations:** 1 Institute of Family Medicine and Public Health, Faculty of Medicine, University of Tartu, Tartu, Estonia; 2 Department of Primary and Community Care, Radboud University Medical Center, Nijmegen, The Netherlands

**Keywords:** Hypertension, Decision aid, Cardiovascular risk, Shared decision making

## Abstract

**Background:**

Coronary heart disease (CHD) mortality among young men is very high and the prevention methods usable in family practice (FP) settings are limited (1,2). The objectives of this study were to investigate the cardiovascular risk profile among young males (18–50) visiting their family doctor (FD) and to find out if using an interactive computer-based decision aid (DA) has advantages in reducing cardiovascular risk factors compared to usual counselling at the FD’s office.

**Methods:**

The study was a cluster-randomized controlled trial including hypertensive male patients aged 18–50 recruited by their FD in 2015–2016. Patients with cardiovascular complications were not included. FDs were randomly divided into intervention groups (n = 9) and control groups (n = 11). Altogether, FDs recruited 130 patients, 77 into the intervention group (IG) and 53 into the control group (CG). IG patients were counselled about cardiovascular risk factors using a computer-based DA. CG patients received usual counselling by their FD. Data was collected with questionnaires, clinical examinations and laboratory analyses at the baseline and at the follow-up visit three months later. We compared the cardiovascular risk factors of the IG and CG patients.

**Results:**

Baseline characteristics of the IG and CG patients were comparable. Of the whole study group, 51.5% (n = 67) of the patients had hypertension grade 1, 45.4% (n = 59) had grade 2 and 3.1% (n = 4) had grade 3. Twenty-seven per cent (n = 21) of the IG and 42% (n = 22) of the CG patients were smokers. We found that shared decision making with the DA was more effective in smoking reduction compared to usual FD counselling: 21 smoking patients in the IG reduced the number of cigarettes per day which is significantly more than the 22 smoking patients in the CG (− 3.82 ± 1.32 (SE Mean) versus + 2.32 ± 1.29; *p* = 0.001). Systolic blood pressure (SBP), diastolic blood pressure (DBP) and the number of cigarettes per day, all showed a statistically significant reduction among patients who were using the DA. Male patients with hypertension grade 2 had a significantly greater reduction in their SBP (− 6.003 ± 2.59 (SE Mean) versus + 1.86 ± 2.58; *p* = 0.038) grade 1. Reduction of DBP, cigarettes per day and CVD risk in general were nearly significant in the IG whereas the CG showed an increase in all of these parameters.

**Conclusion:**

Using interactive DAs at FD’s offices for counselling of young hypertensive male patients is one possibility to help patients understand their risk factors and make changes in their treatment choices. DAs can be more effective in achieving behavioural changes like reducing smoking or blood pressure compared to normal counselling.

## Background

Epidemiological studies have shown that coronary heart disease (CHD) mortality is higher among men before age 75 compared women before age 75. For example, the premature death from CHD per year is approximately 253,000 for men, compared to 77,000 for women before the age of 65 [1]. This large difference between males and females can be explained with a significantly higher presence of cardiovascular risk factors such as systolic blood pressure, total cholesterol and glucose levels as well as smoking among men [2]. Hypertension (HT) in particular has shown a relatively high connection to increased mortality in this study [2].

The need for improvement of cardiovascular disease (CVD) prevention is as important as ever [5], especially in primary care as most of these patients visit their family doctors (FD). Some of the major issues in primary care risk management are lack of time, limited number of educational tools, low patient motivation as well as adherence to the recommended treatment [6]. However, there is evidence that a successful adherence to CVD prevention guidelines and control of risk factors can reduce the mortality significantly [7].

Counselling methods to enhance patients’ motivation and to share information are important topics regarding the possibility that they can influence the health-related behaviour and even reduce the mortality among high-risk populations [8]. These methods include patient education, using motivational interviewing, and shared decision making. *“This is your risk to get a heart attack, what would you be willing to change in your lifestyle now?”.* The latter is the rough idea behind decision aids (DA) that show for patients his or her individual CVD risk, but also a bar chart with the difference if they choose a specific medication, change a habit such as smoking or do nothing. DAs may be a useful tool to be more patient-centred in daily practice and it will not necessarily affect the length of consultation time [9].

Unfortunately, there are no clear answers on the effectiveness of a DA and which kind of DA method should be used. We could find clinically significant results for positive DA effects with high CVD risk patients in a randomized trial with a large sample, but it was not specified for the young male patients suffering from HT [10]. The results in a systematic review investigating the decision support systems and changes in CVD risk factors were not convincing for changes in morbidity or blood pressure and lipid outcome values [11]. Nonetheless, the authors of that study found some studies showing an improvement in mortality and health behaviour changes such as smoking and physical activity. The results may vary because of problems such as poor integration into clinical workflow and failure to promote personalised care or shared decision making. In summary, the study results were inconsistent and again not specified for the population mostly at risk. Besides the literature about decision support systems, there is also poor evidence about the use of shared decision making among patients with HT. The reason for this is that the amount of related studies is low, and the risk of bias was high or could not be determined [12].

This all refers to the need to study the specific effects of DAs in the young male population suffering from HT.

The aims of this study were to: 1) investigate the cardiovascular risk factors profile among young men (18–50) with HT in family practices; 2) analyse the effectiveness of a computer-based DA promoting shared decision making in changing cardiovascular risk factors.

## Methods

This cluster randomised controlled study was conducted in 2015–2016. We sent an invitation to participate in this study to the FD’s mail list, which covers about 95% (881/921) of acting FDs across Estonia. Altogether, 20 family practice centres showed interest in participating in the study. We divided the family practices randomly (a person who was not involved and familiar with the study flipped a coin) into two groups: control group (n = 11) and intervention group (n = 9).

The Ethics Committee of the University of Tartu has approved this study on 22.11.2010, document number 198T-2.

### Participants

The FDs engaged male patients in the age group 18–50 years who had a confirmed diagnosis of hypertension and were prescribed blood pressure medications. Patients with already diagnosed CVDs (e.g. stenocardia, myocardial infarction, atrial fibrillation, vascular diseases of the brain, atherosclerosis, renal failure, and diabetes) were excluded, because our intention was to focus on the most potential group of patients for prevention. Therefore, the younger age group (18–50 years) of males was selected.

### Procedure

Written informed consent was obtained from all subjects before participation in the study. Data for this study was collected with questionnaires, clinical examinations and laboratory analyses. Smoking status and family history about previous cardiovascular events was asked from patients. Patients’ weight, height, waist and blood pressure was measured at the family practice centre. Laboratory analyses for total cholesterol level, low-density lipoproteins, high-density lipoproteins, triglycerides, serum creatinine level, glomerular filtration and urine albumin/creatinine rate were taken and an electrocardiogram was registered by the FDs. Patients were fasting before the laboratory tests. Cardiovascular morbidity risk was calculated by the DA program. FDs used their usual laboratories because their everyday treatment decisions are based on these values.

Prescribed blood pressure medications were registered and information about the purchase from the pharmacy was checked using data from the Estonian Health Insurance Fund.

The data was collected two times, at baseline and at the follow-up visit three months later.

### Study design

The computer-based DA program ‘ARRIBA HERZ’ with interactive visual prompts was used for the intervention [13]. The program was obtained by personal contacts with the researchers and program creators from Marburg, Düsseldorf and Rostock University. The program was translated into Estonian. As Estonian CVD morbidity rates were not available, the European data from the original ARRIBA program was used. Cardiovascular risk by the program is calculated taking into account patient gender, age, smoking status, family history of heart attack or stroke, patient previous cardiovascular events, prescribed blood pressure medication, systolic blood pressure, total cholesterol and high-density lipoprotein-cholesterol levels.

Our DA program calculated morbidity risk for heart attack and stroke for 10 years based on the Framingham algorithm. We categorised patients into two cardiovascular risk groups: low (0–10%) and high risk (> 10%) [14]. Based on NICE recommendations, a 10-year CVD risk more than 20% has been considered as high [15]. Later recommendations suggest offering primary prevention of CVD to all patients who have a 10% or greater risk of developing cardiovascular disease in 10 years [15].

All FDs in the intervention group received four-hour practical training about the use of the DA programme and shared decision making counselling processes. The program generated individualised cardiovascular risk calculations, visual figures of risk summaries and suggestions for lifestyle interventions for each patient, which was used for intervention group patients during counselling. The control group FDs used their usual type of counselling, they did not use any DA or other visual aids. Control group FDs did not receive pre-training about shared decision making. Doctors in both groups consulted the patients according to the schedule as in usual practice and all patients received the necessary treatment.

### Outcome values

We used seven outcome values to analyse the effect of the DA: systolic blood pressure (SBP), diastolic blood pressure (DBP), total cholesterol (TC), body mass index (BMI), waist circumference, cardiovascular disease (CVD) risk calculated by the DA and the number of cigarettes per day of the smokers in the group (including patients who quit or start smoking during the follow-up).

### Statistical analysis

All statistical analyses were performed using SPSS Version 26. Baseline characteristics were compared between the two groups using an Independent-Samples T-test for the mean values and a Contingency Table Analysis including Chi-squared tests for proportions. We checked the distribution pattern of all outcome variables with histograms from a chart builder.

We found an important amount of missing values from the second or third measurement after the follow-up period. A missing value for the second cholesterol and CVD risk measurement was found in 15 patients, or 11.5% of the study sample. A missing value for the second measurement of BMI, waist circumference, SBP and DBP was found in 14 patients, or 10.7% of the study sample. The missing value for the number of cigarettes per day was calculated only with smoking patients. Therefore, we had 2 missing values which was 4.7% of the smoking study sample (n = 43). The missing values were more prevalent in the IG. Therefore, the IG had 12 of the 15 missing values for cholesterol and CVD risk, 10 of the 14 missing values for BMI, waist circumference, SBP and DBP and 2 of the 2 missing values for cigarettes per day.

Little’s Missing Completely at Random (MCAR) Test showed that there is a statistically significant missing pattern (*p* = 0.007). Further analyses clearly showed that this missing pattern is due to dropout, because of patients who did not undergo the second measurements.

This could lead to informative missingness and the risk of bias was increased. On the basis of a negative MCAR test and a high number of missing values, we decided to reduce the risk of bias with a multiple imputation (regression method) of the missing values from the 15 involved patients. The amount of imputations was 20.

Change in outcome measurements before and after three months were tested with a Paired-Samples T-Test. The comparison of changes between the intervention and control groups was conducted using Independent-Samples T-tests. After that we divided patients accordingly into different blood pressure grades and did the comparison of changes between the intervention and control groups only for patients with hypertension grade 2. We used the common classification of hypertension grade 1, 2 and 3 [16]. Again, Paired-Samples T-tests and Independent-Samples T-tests were used.

All *p *values were two sided, and p < 0.05 was defined as statistically significant.

## Results

### Baseline characteristics of the whole study group

During the study period, altogether 130 men were recruited by their own FD to participate in the study, 77 of them were in the intervention group (IG) and 53 in the control group (CG). Table 1 presents the general characteristics of the study group. There are two statistically significant differences between the groups: the mean age in the IG was 3.98 years higher than in the CG (40.96 ± 7.22 (SD) and 36.98 ± 7.94, respectively, *p* = 0.004) and the mean total cholesterol levels were 0.37 mmol/L higher in the IG than in the CG (5.75 ± 1.06 (SD) versus 5.38 ± 0.98, *p* = 0.045). In addition, there was a notable difference in family history for heart attack or stroke: subjects in the IG reported the presence of heart attack or stroke in the family more often than patients in the CG (74% ± 44.5 versus 58% ± 0.5 (SD), (*p* = 0.076), but it was not statistically significant. Twenty-seven percent (n = 21) of the IG and 42% (n = 22) of the CG were smokers. There were also e-cigarette users in both groups, but the amount was very small (five patients in total). However, we did not include them for the smoking frequency analyses, because we had no data about the amount that e-cigarette users are smoking.

**Table 1:** Baseline Characteristics in Intervention vs. Control group

	Intervention group (n = 77)	Control group (n = 53)	*p *value
Age (y)	40.96 (7.22)	36.98 (7.93)	0.004*
Duration of hypertension (y)^†^	10.91 (6.59)	9.92 (6.68)	0.407
Systolic blood pressure (mmHg)	138.47 (12.25)	139.38 (15.49)	0.71
Diastolic blood pressure (mmHg)	89.60 (9.14)	86.49 (9.81)	0.067
BMI (kg/m^2^)	31.59 (5.49)	30.09 (5.7)	0.134
Waist (cm)	107.52 (14.06)	103.23 (14.42)	0.093
Hypertension related medications used (total number)^†††^	1.51 (0.81)	1.57 (0.93)	0.732
Smokers, n (%)	21 (27)	22 (42)	0.362
E-Cigarette users, n (%)	1 (1)	2 (4)	0.250
Cigarettes per day (total number)^††^	16.55 (7.22)	15.05 (8.27)	0.332
Family anamnesis with infarction/stroke, n (%)	45 (58)	39 (74)	0.094
Microalbuminuria diagnosis, n (%)^†††^	3 (4)	2 (4)	0.855
*Laboratory parameters*			
Total cholesterol (mmol/L)^†††^	5.75 (1.06)	5.38 (0.98)	0.045*
High-density lipoprotein (mmol/L)^†††^	1.28 (0.37)	1.29 (0.46)	0.873
Low-density lipoprotein (mmol/L)^§^	3.68 (1.1)	3.42 (0.92)	0.170
Triglycerides (mmol/L)^†††^	1.94 (1.26)	1.83 (1.13)	0.608
Blood glucose level (mmol/L)^§§§^	5.5 (0.57)	5.28 (1.03)	0.116
eGFR (mL/min/1.73m^2^)^†††^	99.83 (16.34)	102.77 (14.16)	0.291
Serum creatinine (mg/dL)^†††^	78.61 (10.42)	77.74 (9.71)	0.633
*Cardiovascular disease risk scores*			
10-year stroke or heart attack risk from decision aid(percentage)^§§^	8.25 (0.08)	6.68 (0.08)	0.293
QRISK-lifetime cardiovascular risk (percentage)	61.73 (13.58)	62.38 (14.59)	0.796

Continuous variables are presented as mean and standard deviation (SD) unless indicated as percentage

BMI—body mass index kg/m^2^; CG—Control Group; IG—Intervention Group

^*^p values under 0.05 are considered as significant and tagged with an asterisk

^†^IG n = 77, CG n = 52

^††^Including patients who started or quit smoking during the follow-up period; IG n = 21, CG n = 22

^†††^IG n = 76, CG n = 53

^§^IG n = 75, CG n = 53

^§§^IG n = 75, CG n = 51

^§§§^IG n = 74, CG n = 53

### Baseline characteristics of patients based on patient’s hypertension grade

We performed the analysis to compare the intervention and control group patients results according to their hypertension grades (Table 2). The patients' division according to their hypertension grades were reported by FDs based on the patients’ blood pressure grade before entering this study. Therefore, it showed the prior blood pressure grades without treatment on the day of the diagnosis. We did not present the hypertension grade 3 because of a very small sample size (n = 4). The IG and CG of hypertension grade 2 patients had similar baseline characteristics and were comparable. Therefore, we conducted further statistical testing only for hypertension grade 2 patients after we made a general comparison between the IG and CG.

**Table 2:** Baseline characteristics in Intervention vs. Control group and divided into Hypertension grades

	Hypertension grade 1			Hypertension grade 2		
	Intervention group (n = 40)	Control group (n = 27)	*p *value	Intervention group (n = 35)	Control group (n = 24)	*p *value
Age (y)	40.15 (7.88)	35.52 (8.4)	0.025*	41.89 (6.61)	38.29 (7.55)	0.058
Duration of hypertension (y)†	10.83 (7.45)	9.58 (8.21)	0.525	10.86 (5.64)	10.5 (4.85)	0.801
Systolic blood pressure (mmHg)	135.37 (11.64)	142.11 (16.12)	0.051	140.77 (10.6)	135.42 (14.32)	0.104
Diastolic blood pressure (mmHg)	87.1 (6.93)	85.19 (9.78)	0.351	91.57 (10.23)	88.08 (10.12)	0.202
BMI (kg/m^2^)	30.08 (3.9)	27.7 (4.23)	0.021*	32.84 (6.43)	32.2 (5.9)	0.7
Waist (cm)	104.1 (11.34)	97.5 (12.62)	0.029*	110.5 (15.96)	108.6 (14)	0.639
Hypertension related medications used (total number)^†††^	1.31 (0.61)	1.63 (1.04)	0.12	1.71 (0.93)	1.5 (0.83)	0.367
Smokers, n (%)	9 (23)	10 (37)	0.27	12 (34)	9 (38)	1.000
E-Cigarette users, n (%)	0 (0)	0 (0)	1.000	1 (3)	2 (8)	0.561
Cigarettes per day (total number)^††^	15.78 (4.381)	17.4 (9.06)	0.210	17.67 (8.94)	15.22 (4.52)	0.887
Family anamnesis with infarction/stroke, n (%)	22 (55)	19 (70)	0.307	22 (63)	18 (75)	0.402
Microalbuminuria diagnosis, n (%)^†††^	2 (5)	1 (4)	1.000	2 (6)	1 (4)	1.000
*Laboratory parameters*						
Total cholesterol (mmol/L)^†††^	5.79 (1.02)	5.2 (0.9)	0.017*	5.69 (1.14)	5.48 (1.01)	0.463
High-density lipoprotein (mmol/L)^†††^	1.23 (0.29)	1.41 (0.54)	0.084	1.33 (0.45)	1.19 (0.32)	0.196
Low-density lipoprotein (mmol/L)^§^	3.71 (0.86)	3.16 (0.79)	0.012*	3.59 (1.33)	3.63 (0.99)	0.916
Triglycerides (mmol/L)^†††^	2 (1.46)	1.56 (0.92)	0.174	1.88 (1.06)	2.02 (1.18)	0.638
Blood glucose level (mmol/L)^§§^	5.48 (0.67)	5.22 (1.29)	0.298	5.52 (0.46)	5.28 (0.69)	0.117
eGFR (mL/min/1.73m^2^)^†††^	97.04 (14.09)	102.49 (12.43)	0.110	102.75 (18.62)	104.6 (15.24)	0.689
Serum creatinine (mg/dL)^†††^	80.26 (9.99)	77.86 (8.19)	0.306	76.86 (11)	76.25 (9.88)	0.829
*Cardiovascular disease risk scores*						
10-year stroke or heart attack risk from decision aid(percentage)	9.59 (9.04)	9.2 (8.61)	0.349	7.87 (9.37)	8.43 (10.27)	0.462
QRISK-lifetime cardiovascular risk (percentage)	60.4 (13.9)	57.8 (13.9)	0.454	62.82 (13.27)	65.42 (13.2)	0.367

Continuous variables are presented as mean and standard deviation (SD) unless indicated as percentage

BMI—body mass index kg/m2; CG—control group; IG—intervention group

^*^p values under 0.05 are considered as significant and tagged with an asterisk and bold

^†^Hypertension grade 1: IG n = 40, CG n = 26

Hypertension grade 2: IG n = 35, CG n = 24

^††^including patients who started or quit smoking during the follow-up period; Hypertension grade 1: IG n = 9, CG n = 10 Hypertension grade 2: IG n = 12, CG n = 9

^†††^Hypertension grade 1: IG n = 39, CG n = 27

Hypertension grade 2: IG n = 35, CG n = 24

^§^Hypertension grade 1: IG n = 38, CG n = 27

Hypertension grade 2: IG n = 35, CG n = 24

^§§^Hypertension grade 1: IG n = 37, CG n = 27

Hypertension grade 2: IG n = 35, CG n = 24

### Outcome changes of the whole group

In comparing IG and CG patients’ cardiovascular risk factors at the follow-up visit, we found a statistically significant change in smoking in the IG group (Table 3). The IG smoking patients (n = 21), reduced the number of cigarettes per day significantly more than the smoking patients in the CG (n = 22) (− 3.82 ± 1.32 (SE Mean) versus + 2.32 ± 1.29; *p* = 0.001). The IG patients (n = 77) had a greater reduction in DBP than the CG patients (n = 53) with a clear tendency to become significant (− 2.96 ± 1.27 (SE Mean) versus − 0.4 ± 1.41; *p* = 0.180). The difference in reduction of other outcome values between IG and CG were statistically and clinically not significant. However, we had a statistically significant reduction over time only in the IG for SBP (− 3.2; *p* = 0.039), DBP (− 2.96; *p* = 0.02) and number of cigarettes per day (− 3.82;* p* = 0.004).

**Table 3:** Changes in cardiovascular risk factors after 3 months

	Intervention group at baseline	Intervention group after 3 months	Intervention group mean difference over time (*p *value)	Control group baseline	Control group after 3 months	Control group mean difference over time (*p *value)	Mean difference of change between IG and CG (*p *value)
Systolic blood pressure (mmHg)^†^	138.47 (1.4)	135.27 (1.76)	− 3.2 (0.039)*	139.38 (2.13)	137.66 (1.64)	− 1.72 (0.366)	− 1.48 (0.543)
Diastolic blood pressure (mmHg)^†^	89.6 (1.04)	86.63 (1.16)	− 2.96 (0.020)*	86.49 (1.35)	86.09 (1.38)	− 0.4 (0.777)	− 2.56 (0.180)
Total cholesterol (mmol/L)^†^	5.76 (0.12)	5.67 (0.14)	− 0.1 (0.344)	5.38 (0.13)	5.27 (0.15)	− 0.11 (0.280)	+ 0.01 (0.924)
BMI (kg/m^2^)^†^	31.59 (0.63)	31.59 (0.66)	+ 0.01 (0.967)	30.09 (0.78)	30.08 (0.79)	− 0.01(0.976)	+ 0.01 (0.962)
Waist (cm)^†^	107.52 (1.6)	107.44 (1.62)	− 0.08 (0.852)	103.23 (1.98)	103.66 (2.03)	+ 0.43 (0.473)	− 0.51 (0.475)
Cigarettes per day (total number)^††^	16.86 (1.58)	13.04 (1.46)	− 3.82 (0.004)*	15.05 (1.76)	17.27 (1.99)	+ 2.23 (0.085)	− 6.05 (0.001)*
10-year stroke or heart attack risk from decision aid(percentage)	8.48 (0.94)	7.83 (0.97)	− 0.65 (0.182)	7.96 (1.43)	7.89 (1.51)	− 0.08 (0.883)	− 0.57 (0.429)
QRISK-lifetime cardiovascular risk (percentage)	61.73 (1.5)	61.35 (1.6)	− 0.38 (0.571)	62.38 (2)	61.7 (2)	− 0.68 (0.400)	+ 0.3 (0.773)

Continuous variables are presented as mean and standard error of mean (SE MEAN), unless indicated as *p *value

BMI—body mass index kg/m2; CG—Control Group; IG—Intervention Group

^*^p values under 0.05 are considered as significant and tagged with an asterisk and bold

^†^IG n = 35; CG n = 24 (with multiple imputation regression method)

^††^Including patients who started or quit smoking during the follow-up period; IG n = 21; CG n = 22

### Outcome changes of patients with hypertension grade 2

There was a significantly greater SBP reduction in the hypertension grade of 2 patients in the IG. (Table 4). Moreover, we found a nearly significantly greater DBP, cigarettes per day and CVD risk reduction in the IG whereas the CG showed an increase in all of these parameters.

**Table 4:** Changes in Cardiovascular Risk Factors of Patients with Hypertension Grade 2 after 3 months

	Intervention group at baseline	Intervention group after 3 months	Intervention group mean difference over time (*p *value)	Control group baseline	Control group after 3 months	Control group mean difference over time (*p *value)	Mean difference of change between IG and CG (*p *value)
Systolic blood pressure (mmHg)^†^	140.77 (1.79)	134.77 (2.42)	− 6 (0.021)*	135.42 (2.92)	137.27 (2.37)	+ 1.86(0.471)	− 7.86 (0.038)*
Diastolic blood pressure (mmHg)^†^	91.57 (1.73)	87.39 (1.65)	− 4.18 (0.056)	88.08 (2.06)	88.77 (1.99)	+ 0.69 (0.730)	− 4.87 (0.119)
Total cholesterol (mmol/L)^†^	5.69 (0.19)	5.59 (0.23)	− 0.09 (0.540)	5.48 (0.21)	5.39 (0.23)	− 0.09 (0.594)	− 0.01 (0.985)
BMI (kg/m^2^)^†^	32.84 (1.09)	32.99 (1.15)	+ 0.15 (0.627)	32.2 (1.2)	32.15 (1.24)	− 0.05 (0.792)	+ 0.2 (0.617)
Waist (cm)^†^	110.49 (2.7)	110.17 (2.81)	− 0.31 (0.579)	108.58 (2.87)	108.65 (3.08)	+ 0.06 (0.947)	− 0.38 (0.721)
Cigarettes per day (total number)^††^	17.67 (2.58	14.2 (2.18)	− 3.47 (0.069)	13.7 (2.03)	15 (1.49)	+ 1.3 (0.471)	− 4.77 (0.073)
10-year stroke or heart attack risk from decision aid(percentage)	9.59 (1.53)	8.7 (1.49)	− 0.89 (0.214)	7.87 (1.91)	8.13 (2.09)	+ 0.26 (0.753)	− 1.15 (0.287)
QRISK-lifetime cardiovascular risk (percentage)	62.82 (2.24)	62.1 (2.42)	− 0.71 (0.445)	65.42 (2.7)	65.44 (2.9)	+ 0.02 (0.985)	− 0.69 (0.585)

Continuous variables are presented as mean and standard error of mean (SE MEAN)

BMI—body mass index kg/m2; CG—Control Group; IG—Intervention Group

^*^p values under 0.05 are considered as significant and tagged with an asterisk

^†^IG n = 35; CG n = 24 (with multiple imputation regression method)

^††^including patients who started or quit smoking during the follow-up period; IG n = 12; CG n = 10

**Fig. 1:**
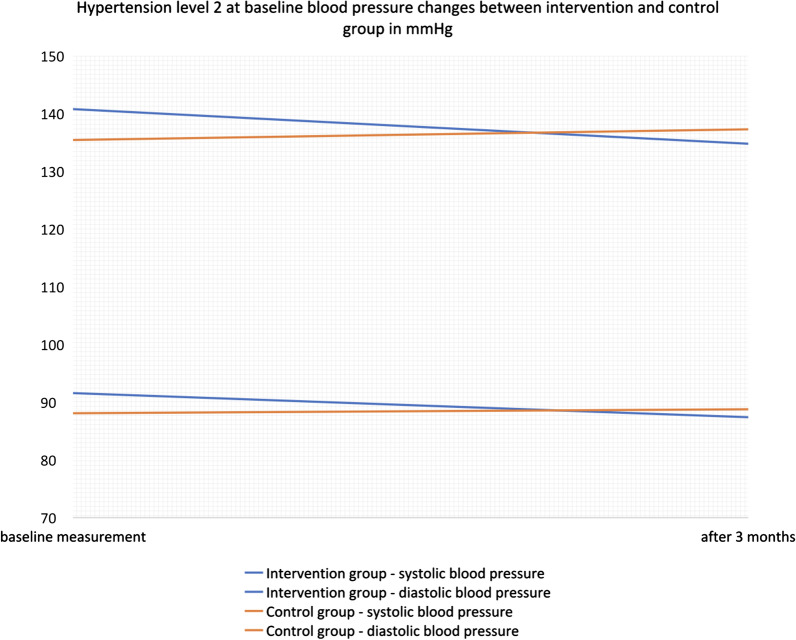
Changes in blood pressure of patients that were diagnosed with hypertension grade 2

Figure 1 shows clinically important blood pressure changes of both groups. To be specific, the 35 patients of the IG had a significantly greater SBP reduction than the 24 patients of the CG (− 6.003 ± 2.59 (SE Mean) versus + 1.86 ± 2.58; *p* = 0.038). The mean reduction among IG patients (n = 35) was also greater than in the CG patients (n = 23) for DBP (− 4.18 ± 2.19 (SE Mean) versus + 0.69 ± 1.99; *p* = 0.119) and CVD risk (− 0.89 ± 0.71 (SE Mean) versus + 0.26 ± 0.84; *p* = 0.287). Additionally, the number of cigarettes per day in the 12 smoking patients of the IG also had a greater reduction than the 10 smoking patients of the CG (− 3.47 ± 1.91 (SE Mean) versus + 1.3 ± 1.8; *p* = 0.073). The mean reduction over time was only significant for SBP (− 6.003; *p* = 0.021) in the IG.

## Discussion

This study demonstrates that use of the DA in the FD consultation helps male patients with hypertension reduce the number of cigarettes. Furthermore, we found a statistically significant drop of the systolic and diastolic blood pressure during three months. An equally important finding was the statistically and clinically significant blood pressure decrease in the intervention group patients with hypertension grade 2.

These changes may be related to better medication adherence or modifications in lifestyle. In any case, the most important is the change of the patients’ cardiovascular risk factors in general. Despite our patients not quitting smoking, the reduction in numbers of cigarettes per day is a very important outcome, because it refers to behavioural change which is often hard to achieve. We are aware that a reduction in the number of cigarettes is not enough to reduce the cardiovascular risk in total, but the behavioural readiness for change was clear. Most of the patients need additional support and counselling to move forward because smoking is one of the leading causes of preventable mortality worldwide and the relation to life expectancy is even higher for men [17].

The significantly greater systolic blood pressure decrease was found only in the hypertension grade 2. However, hypertension grade 2 increases cardiovascular risk significantly, therefore these patients may benefit more from treatment interventions than hypertension grade 1 patients. High blood pressure is also one of the main modifiable cardiovascular risk factors among young male [18]. We did not receive statistically significant changes in the other measurements, but some other factors showed a greater tendency to become clinically and statistically significant in the intervention group compared to the control.

We did not obtain statistically significant 10-year risk for heart attack or stroke differences between intervention and control groups. Most of the generally used cardiovascular risk scores are not suitable for younger patients because they calculate only the 10-year heart attack and stroke risk. A good CVD risk calculation for younger patients could give us more significant results in prevention. Modifying the risk factors as early as possible was also the idea behind our study.

Previous studies of the Estonian population, the country of this research, have shown a high prevalence of CVD risk factors between the age 20–65 [3, 4]. High blood pressure was found to be the main abnormality after abdominal obesity and both risk factors showed a strong male predominance. Moreover, the awareness of high blood pressure levels was the lowest among men aged 20–39 [19]. Metabolic syndrome also seemed to have a relatively higher prevalence among young Estonian men when compared to other European countries [20].

Multidisciplinary studies influencing lifestyle habits for hypertension have had some positive effects, but some interventions take a lot of time and are not eligible for general practice [21]. Therefore, our study showed how the integration of DA into FDs consultation is possible and can be effective in inducing behaviour change and reduction of risk factors. Previous studies have reported that using a DA can lead to better informed patients [22] and have little or no effect on visit duration [9]. The utilisation of personal data also gives personal solutions and a better understanding how each step will have an influence on the patient’s future health. We suspect a positive effect on the use of shared decision making and patient education which lead to a reduction in cardiovascular risk factors. However, further studies are needed.

The strengths of this study were the use of a DA that covers important key features of successful decision support systems. These key features included a computer-based support, giving assessments and recommendations, linking with electronic patient charts, promoting action and provision of decision support at the time and location of patient contact. These key features are from the recommendation of a systematic review of clinical decision support systems in CVD prevention [11]. Furthermore, we used a DA with visual bar charts for individual CVD risk and the differences when choosing a specific treatment plan. This promoted the shared decision making between doctors and patients. In Estonia, all patients diagnosed with type 2 diabetes or hypertension are part of yearly follow up and FD’s are reimbursed according to the “Pay for Performance” system, therefore the implementation into clinic workflow was not an issue. We conducted an accurate measurement of the baseline characteristics and can describe the comparability of the groups very well. The participating family practices were divided randomly into intervention and control groups.

However, this study also had some limitations. One of the main limitations was a small sample size, especially after dividing the sample into hypertension grade 1 or 2. The statistical comparison could have more significant results, if the sample size would have been larger. The latter was related to the limited study period. The adaptation and translation of the DA program into Estonian took longer than we expected. As we received funding only for three years the recruitment time was limited.

In addition, there was a relatively high number of missing data. Fifteen second measurements for the outcome values were missing and this affects 11.5% of our sample. As we described in the methods section, the missing values are not MCAR and due to dropout of patients who did not go to the second measurements. The reasons for that were most probably a lack of time or interest. This could influence the study results, especially because patients who are motivated enough to change their own lifestyle will more probably show up for the second or third examination.

The IG had more dropouts (the exact numbers are described in the methods section), so there is a chance that we did not measure the outcome values of some less motivated patients and the risk for selection bias was increased. On the other hand, a group with less dropouts could be more motivated on average. The numbers are described in the methods section, and luckily we had only 2 dropouts for the 43 smoking patients. This made up only 4.7% of the study sample used in the statistical comparison for change in cigarettes per day, so our main result had the least impact from missing values and the associated bias. Nevertheless, we conducted the multiple imputation (regression method) and this could reduce the risk of missing data bias substantially [23].

The follow-up was only 3 months, therefore we do not have evidence for the long-term effects of a DA. In our outcome values we did not have a second measurement of LDL-cholesterol and this is a very important factor regarding CVD risk. There are new DAs that include genetic data. This may improve the outcomes, however we found evidence that genetic risk communication alone does not affect preventative lifestyle modification [24]. Still, there could be a positive effect of genetic risk communication in combination with a DA. Furthermore, the smoking frequency of the patients was self-reported, and a minority of smokers could have a day-to-day variability in the number of cigarettes [25].

Future studies are needed to investigate this research question with larger sample sizes, longer observation periods and a CVD risk calculation for younger patients.

## Conclusions

Using interactive DAs at FD’s offices for the counselling of young hypertensive male patients is one possibility to help patients understand their risk factors and make changes in their treatment choices. DAs can be more effective in achieving behavioural changes like reducing smoking or blood pressure compared to normal counselling in young hypertensive male patients.

## Data Availability

The datasets used and/or analysed during the current study are available from the corresponding author on reasonable request.

## Abbreviations

BMI: Body mass index
CG: Control group
CHD: Coronary heart disease
CVD: Cardiovascular disease
DA: Decision aid
DBP: Diastolic blood pressure
FD: Family doctor
HT: Hypertension
IG: Intervention group
SBP: Systolic blood pressure
TC: Total cholesterol

## Declarations

### Standards of reporting

Our study adheres to CONSORT guidelines.
